# A Proposal of Remedies for Oral Diseases Caused by Candida: A Mini Review

**DOI:** 10.3389/fmicb.2018.01522

**Published:** 2018-07-09

**Authors:** Tomoko Ohshima, Satoshi Ikawa, Katsuhisa Kitano, Nobuko Maeda

**Affiliations:** ^1^School of Dental Medicine, Tsurumi University, Yokohama, Japan; ^2^School of Engineering, Osaka University, Suita, Japan; ^3^Technology Research Institute of Osaka Prefecture, Osaka, Japan

**Keywords:** Candida attachment, biofilm formation, denture plaque, atmospheric plasmas, plasma-treated water, sterilization, antifungal molecules

## Abstract

An opportunistic pathogen, Candida is not only related to oral problems such as oral candidiasis and denture stomatitis, but also to systemic diseases such as aspiration pneumonia and fungemia. The carriage rate of Candida species in the oral cavity of individuals wearing dentures and with removable orthodontic appliances, has increased. Moreover, it is one of the causal pathogens in refractory infected root canals because of its resistance to antifungal drugs in root canal therapy and poses a great challenge during the treatment of patients. This problem has led to the search for alternative strategies for the treatment and management of *C. albicans* infections. In this mini review, recent preventive strategies against Candida infection in the oral mucosa with natural product-derived antifungal molecules were discussed. Inhibitory strategies by introducing competitive naturally-derived antifungal peptide molecules with Candida adhesion molecules were specifically introduced. In addition, novel sterilization methods for Candida-infected root canals and tooth structures in the oral cavity were considered, with focused attention on the activities of reactive oxygen species. The possibility of application of these novel strategies in clinical treatments and daily life was also proposed.

## Introduction

*Candida* includes opportunistic pathogens that cause infections in immunocompromised hosts, as well as in healthy hosts with altered microbiota (also referred to as “dysbiosis”). Among the various human fungal pathogens, *Candida albicans* accounts for the majority of infections, followed by *C. glabrata* (Rex et al., 1999). These opportunistic fungi cause substantial problems because of their resistance to most antifungal drugs (Wisplinghoff et al., 2014). This problem has led to the search for alternative strategies for the treatment and management of *C. albicans* infections. Furthermore, *C. albicans* is well known as a biphasic fungus that grows in two different forms, yeast and hypha (Odds, 1988). When *C. albicans* exists on the mucous membrane or on the skin surface, it lives in the yeast form, but when it invades the tissues, it often takes the hyphal form that possesses stronger pathogenicity than that of the yeast form (Odds, 1988). *C. albicans* biofilm comprises a mixed state of yeast and hyphal forms (Andes et al., 2004). The pathogenicity of biofilm is much greater than that of the planktonic state, with expression of virulence factors including resistance not only to antifungals, but also host phagocytosis (Nett et al., 2010; Rajendran et al., 2010; Williams and Lewis, 2011). Moreover, mixed-species biofilm is significantly more invasive compared to single fungus or single bacterial biofilms (Cavalcanti et al., 2015). It has been estimated by the National Institute of Health that more than 80% of infection diseases are caused by pathogens in biofilms (Harriott and Noverr, 2011). Therefore, inhibition strategies against *Candida* biofilms are more urgent.

Some candidates to control *Candida* biofilm have been proposed, based on the nature of *Candida* colonization and growth, to disrupt or remove them from the oral cavity. The two most frequent oral diseases associated with *Candida* are denture stomatitis (Arendorf and Walker, 1987; Gendreau and Loewy, 2011) and refractory root canal infection (Najzer-Felger et al., 1992; Molander et al., 1998; Waltimo et al., 1999). In this mini review, new strategies to overcome these *Candida* diseases in the oral cavity have been introduced and discussed.

## Candida biofilm on dental materials and its role as a Candida reservoir

Colonization of microorganisms on denture base materials occurs mainly because of the strong adherence of *Candida* species to the acrylic resin base (Nalbant et al., 2008). The direct adhesion of *Candida* species to denture surfaces is the essential pathogenic factor in denture stomatitis. Because *Candida* co-aggregates with several plaque bacteria and makes a strict biofilm on dentures, the latter has been suggested as a reservoir of plaque and affects the systemic health of denture-wearers (Nikawa et al., 1998; Gendreau and Loewy, 2011; Ribeiro et al., 2012). Antifungal therapy is effective for the treatment of acute inflammation associated with denture stomatitis; however, the effectiveness is only short-term, and recurrence is possible shortly after discontinuing treatment or because of the appearance of drug-resistant strains. Porous surfaces and hydrophobicity of acrylic resin for denture bases facilitate the colonization of hyphal forms of *C. albicans* on the surface. *In vitro* studies have shown that hydrophilic coating materials can decrease the adhesion of *C. albicans*, though not that of other *Candida* species (Yoshijima et al., 2010; Gendreau and Loewy, 2011).

Conversely, reports regarding *Candida* and bacterial plaque adhesion on alloy materials have been scarce. From a topographical study of biofilm development, surface roughness and contact angle were shown to be the major factors affecting microbial adherence (Teughels et al., 2006). There have been various reports regarding the effects of surface roughness on plaque adherence (Bollen et al., 1996; Zissis et al., 2000; Teughels et al., 2006). The threshold of surface roughness for plaque accumulation was found to be 0.2 μm (Quirynen and Bollen, 1995; Bollen et al., 1997), although a recent report found the surface roughness of alloy samples to be below 0.05 μm after polishing, and that of the resin was below 0.09 μm; there was no difference before and after salivary coating (Urushibara et al., 2014). Though the contact angle in all samples, without salivary coating, was close to 90°, it was reduced to 35° with salivary coating (Urushibara et al., 2014). This result indicated that the surface of all materials was coated with salivary components and hence displayed close to hydrophilic properties. Among the salivary components, the majority are thought to be proteins because the surface of materials exposed to the oral environment was directly transformed by the spontaneous adsorption of protein-dominated films, as seen in pellicle on enamel (Lamkin et al., 1996; Lendenmann et al., 2000). The amount and composition of this protein seemed to be affected by the amount of biofilm formed. Kolenbrander et al. pointed out that plaque bacteria bound selectively to pellicle protein molecules derived from the salivary coating on the tooth surface (Kolenbrander et al., 2002). Because this phenomenon seemed to affect the degree of biofilm formation, analysis of the composition of the salivary protein attached to each material was considered necessary. In addition, calcium ions contributed to the attachment process involving the alloy surface, salivary protein, and microorganisms (Hanawa and Ota, 1991; Rosan, 1992). In a report addressing the amount of calcium on the surface of alloys by an electron probe microanalyzer (EPMA) (Urushibara et al., 2014), that of gold-copper-platinum alloy (PGA) was greater than that of the other four alloys tested (commercially pure titanium, CP Ti; titanium alloy, Ti 6-7; cobalt-chromium alloy, Co-Cr; and silver-palladium copper-gold alloy, Ag-Pd-Au) and acrylic resin, but the adhesion level of *C. albicans* was shown to follow the order: PGA = CP Ti > Ti 6-7 > Co-Cr > Ag- Pd-Au. The difference in adhesion on different materials appeared to be caused by the variety of salivary proteins adsorbed on the surface, rather than the amount of bound calcium that affected non-selective adhesion. The reason for the lowest adhesion rate on Ag-Pd-Au was likely because of the presence of silver ions (Uchida et al., 2004). Silver (Ag) is an excellent material with anti-microbial properties and lacking any toxicity to the human body (Lansdown, 2006). However, because of the aesthetic disadvantage of silver turning black upon oxidation, its usage in dentistry is limited.

## Prevention of Candida infection in oral mucosa using antifungal molecules

Some researchers have considered the use of microbicidal molecules in the composition of denture materials to prevent denture stomatitis. The modified PMMA denture base, containing a polymeric biocide or a biocide-releasing polymer, exhibited fine antimicrobial properties *in vitro* (Sivakumar et al., 2014); however, they have been incriminated in some cellular studies because of introducing toxic effects. The formulations are being tested, and the implications of antimicrobial macromolecules on health and the environment need to be completely assessed before the products are brought to the market. To avoid this process, some researchers have focused on natural alternatives, without toxic effects to humans and the environment.

### Inhibitory strategy by competition with Candida adhesion molecule

Considering the adhesion mechanism of *Candida*, its major mode of action has been the focus of research. The outermost layer of the *Candida* cell wall is covered with hydrophilic polysaccharides, such as mannan or galactomannan (Shibata and Okawa, 2006; Netea et al., 2008). These mannans function as adhesins on the fungal surface and are involved not only in adhesion to the host cell (Calderone, 1993), but also in the adsorption to plastic plates (Watanabe et al., 1999). The β-1, 2-linked mannooligosaccharide in mannan exhibits strong antigenicity and is associated with the induction of TNF-α, a pro-inflammatory cytokine (Jouault et al., 1995; Shibata and Okawa, 2006). Mannan also promotes wound healing by increasing the proliferation of fibroblasts and vascular endothelial cells, and enhancing the production of collagen (Jettanacheawchankit et al., 2009). Recently, mannan-coated food and medical devices have been developed, and a variety of functions of mannan has been confirmed (Han et al., 2000; Hiragun and Hide, 2011). Based on the findings of these clinical reports, mannan appears to be effective in preventing the development of local and systemic diseases, including denture stomatitis.

Sato et al. conducted research to examine whether mannan coating on the acrylic surfaces of the denture base could inhibit the adhesion of *C. albicans* and *C. glabrata* (Sato et al., 2013). Results showed that mannan-coating significantly inhibited the adhesion of *C. albicans* and *C. glabrata* in a concentration-dependent manner. Overnight coating with 0.1 mg/mL of mannan showed an inhibitory effect on the adhesion of the hyphal form of *C. albicans*, which is a necessary step in forming *Candida* biofilms (Odds, 1988).

There was a similar trial using chitosan, which is a partial de-acetylated chitin, a polysaccharide composed of glucosamine (2-amino-2-deoxy-d-glucose) and *N*-acetyl glucosamine (2-acetamido-2-deoxy-d–glucose) units linked by β (1 → 4) bonds. Chitin is also one of the main components of cell wall, but not an adhesion molecule for *Candida*. Chitosan's antimicrobial activity against a variety of microorganisms, including fungi, is well-established (Costa et al., 2012; Leceta et al., 2013; Upadhyaya et al., 2013). Chitosan inhibited *C. albicans* biofilm adhesion, formation, maturation, and co-aggregation (Costa et al., 2014), although the mechanism is not yet known (Costa et al., 2017). However, chitosan possesses some disadvantages, namely its insolubility in water, high viscosity, tendency to coagulate proteins at high pH (Kumar, 2000; Rabea et al., 2003; Costa et al., 2012), and an allergy-inducing property.

### Inhibition strategy with antifungal peptide molecules derived from natural products

Although fungicidal agents can prevent *Candida* infections directly and extensively, the risk of significant side effects, such as occurrence of resistant species or toxicity to humans and the environment, has also been pointed out. Thousands of anti-microbial peptides have been identified and summarized in several reviews (De Lucca, 2000; Yeaman and Yount, 2003; Bulet et al., 2004; Jenssen et al., 2006), among which, antifungal peptides from ubiquitous plants and animals are of utmost importance because most of them act in the front line of defense against infection, without any toxicity to the host organs. Most antifungal peptides are small cationic peptides with less than 50 amino acids, whose positive charge is essential in binding to the negatively charged membrane or cell wall of fungi. However, those antifungal mechanisms are specific and multifactorial, including stimulatory effects on the human immune system (Matejuk et al., 2010). Matejuk et al. divided antifungal peptides into three groups: (1) primary antifungal peptides (such as glucan synthesis inhibitor and histidine-rich peptides), (2) wide-spectrum antimicrobial peptides (α-helical linear peptides, such as magainin, LL-37, and cyclic di- or oligo-peptides such as defensins), and (3) proteolytic fragments of proteins (such as lactoferrin). However, positive effects of these peptides in clinical trials with dental appliances have been scarce. In general, the intact molecules without any modification are of short-term efficacy. With the technological development of chemosynthesis, antimicrobial functional groups could be introduced into polymeric agents to prolong the life of antifungal activity (Kenawy et al., 2007). Polymerized materials, such as acrylic resin, are fit to apply in dentistry. Trials are continuing for the development of materials applicable to dental and medical devices, as well as for food wrappers or containers (Appendini and Hotchkiss, 2002).

## Sterilization of Candida-infected root canals and tooth structures in the oral cavity

*Candida* is frequently detected in infected root canals with refractory apical periodontitis characterized by persistent percussion sensitivity (Hamaguchi et al., 2002). *C. albicans and C. glabrata* are resistant to various agents, including calcium hydroxide (Ca(OH)_2_), used in root canal therapy (Velera et al., 2001).

The disinfection technique generally used in endodontic therapy involves mechanical cleaning and chemical sterilization. Sodium hypochlorite (NaClO) has traditionally been used as the chemical for this purpose because of its strong sterilization action *in vitro* (Byström and Sundqvist, 1983; Radcliffe et al., 2004). However, it highly irritates human tissues and is associated with a risk of injury caused by extravasation in the periapical tissue, leakage into the oral mucosa, and dispersion to skin and clothes (Bowden et al., 2006; Donald et al., 2008) during dental treatment. Moreover, the success rate has been found to be low because of insufficient effects. A systematic review of literature during 2007 and 2008 pointed out that 20–30% of patients had recurrent symptoms post-treatment. This low rate of success may be attributed to the insufficiency of chemical disinfectants. In addition, strong chemicals involve high risk to humans, as described above. Recently, an ozone-gas-supplying device has been developed, but because of the low water solubility and permeability of ozone, its effects was not sufficient in the human body (Johansson et al., 2009; Almaz and Sönmez, 2015) (Table 1). Moreover, it is also very expensive.

**Table 1:** Properties of microbicidal chemicals.

	**Chemical molecule**	**Microbicidal effect**	**Permiability**	**Effective site**	Oxidationpower^*^ (Redox potential)	**Residual toxicity (life-span)**
Sterilization with chemicals	*O_3_*	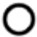	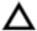	Cell wall/cell membrane	High (2.08V)	Low (−1 h)
	*H_2_O_2_*	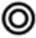	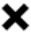	Inside of cell	Intermediate (1.76V)	High (stable)
	*NaCIO*	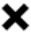	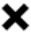	Inside of cell	Intermediate (1.48V)	High (meta–stable)
Sterilization with plasmas	*OH·*	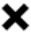	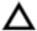	Cell wall/cell membrane	High (2.38v)	No (–μs)
	*$O_{2}^{-}$·*	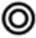	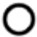	Cell wall/cell membrane	Low (0.645V)	No (−10 s)
	*HOO·*	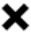	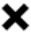	Inside of cell	Intermediate (1.44V)	No (−10 s)

* *Handbook of chemistry: pure chemistry, 5th ed. (The Chemical Society of Japan)*.

For disinfection of teeth, in addition to high microbicidal effects, penetrability to a sufficient depth (at least a few mm), along with low residual toxicity is important. Table 1 summarizes the properties of microbicidal molecules. Among these, H_2_O_2_ is often used in dental treatment because of its low microbicidal effect at working concentrations. Some radicals show high sterilization effects without residual toxicity. HOO· radicals fulfill this requirement quite well. We will, therefore, discuss a sterilizing technique using this molecule, generated by plasma at atmospheric pressure.

### Plasma sterilization efficacy in liquids or humidified phases and the mode of action with radical species

**Figure 1:**
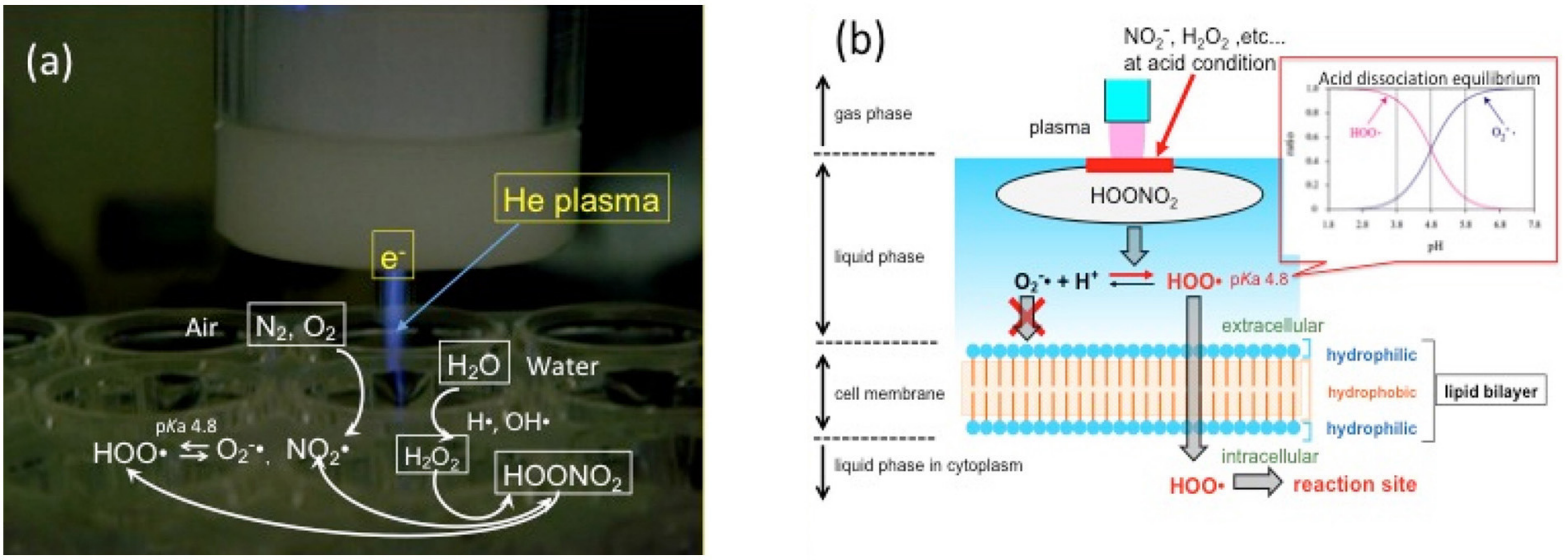
Mode of action of plasma on liquids**. (a)** Chemical species, including nitrous acid and hydrogen peroxide, from air and water are brought together to generate HOONO2, which dissociates into superoxide (O2^−^·) or HOO radical in plasma. HOONO2 is the precursor of HOO·, a key molecule of plasma sterilization**. (b)** The concentration of HOO· is higher than that of O2^−^· at pH < 4.8 because of acid dissociation equilibrium. Uncharged HOO· can easily penetrate cell membranes and introduce oxidation stress inside the cell.

Considering medical and dental applications in the human body, inactivation of pathogens in the liquid or humidified phase is important. In the gas phase, where pathogens can be directly exposed to atmospheric plasma, it is not very difficult to inactivate microorganisms. However, in the liquid phase, it is more difficult because of the barrier of water against plasma. In fact, some researchers concluded that plasma sterilization was less effective than conventional Ca(OH)_2_ or NaClO methods (Pan et al., 2013; Schaudinn et al., 2013). However, this difficulty was overcome by applying the reduced-pH method (Ikawa et al., 2010). The reason for the dramatic sterilization effect by plasma appearing at a low pH can be explained by the property of reactive oxygen species. Oxygen and nitrogen molecules in the air are converted to superoxides (${\text{O}}_{2}^{-}\cdot$) through peroxynitric acid HOONO_2_ by the electron of the plasma in gas phase (Figure 1a) (Ikawa et al., 2016). The ${\text{O}}_{2}^{-}\cdot$ that penetrated into the water is changed into the hydroperoxy radical (HOO·) by acid dissociation equilibrium as follows:

$$\begin{array}{r} HOO\cdot ⇄O_{2}^{-}\cdot +H^{+}\;\left(p\text{K}a=4.8\right) \end{array}$$

The uncharged radical HOO· can easily penetrate into cell membranes with strong microbicidal activity (Korshunov and Imlay, 2002; Takai et al., 2013) (Figure 1b).

From the evaluation test by Yamazaki et al., the sterilization efficacy in liquid phase by the reduced-pH method, using a suspension of *C. albicans*, was not enough at pH > 4.5; although the counts of remaining living *Candida* cells were significantly reduced below the detection limit at pH 3.5 (Yamazaki et al., 2011).

### Sterilization effects of plasma-treated water (PTW) on root canals infected by Candida species

To evaluate the efficacy of plasma sterilization in infected root canals, Yamamoto et al. prepared *in vitro* infection models using *Candida* species (Yamamoto et al., 2017). The infected root canal filled with a pH 3.5 buffer was irradiated with plasma, the remaining fungi were collected with a paper point, and cultured in broth for 48 h for a turbidity test, mimicking a clinical evaluation test. Most of the cultured samples were turbid, and an adequate sterilizing effect could not be confirmed. A possible reason for this could have been the low diffusion or convection of gas and liquid in the narrow, deep area of the root canal, which restricted the HOO· supply into the area. As a remedy, they focused on plasma-treated water (PTW), in which water was exposed to plasma to accumulate active species and maintain microbicidal activity for several minutes at room temperature (Ikawa et al., 2016). This was anticipated to contain the generated radicals that were delivered to the deep area. The procedure to generate PTW was described in some earlier reports (Tasaki et al., 2017; Yamamoto et al., 2017). The minimum inhibitory concentration (MIC) of PTW determined against *C. albicans* and *C. glabrata* was a dilution ratio of 0.25 (Yamamoto et al., 2017). The efficacy of PTW for endodontic sterilization by *in vitro* test using the reduced-pH method was confirmed in the report. No growth of residual fungi was seen in any of the infected root canal models of *C. albicans* and *C. glabrata* (Yamamoto et al., 2017). PTW has a strong microbicidal activity and a short half-life of the active species in it, suggesting that it is a very safe root canal irrigant with low residual activity. The inherent biological safety of atmospheric plasma has been noted (Delben et al., 2016). The property of PTW, by which it detoxifies within a short period of time, is not present in conventional disinfectants, and hence is highly advantageous as a disinfectant applicable to living microorganisms.

## Conclusion

A remedy for oral diseases caused by *Candida* has not yet been established. In this review, potential proposals for remedies for the oral cavity were discussed by focusing on methods with competing molecules against *Candida* and sterilizing radical species generated by plasma technics, with low tolerance rates in *Candida* and low residual toxicity for human tissues. These novel strategies will overcome the *Candida* problems in the oral cavity in the near future.

## Author contributions

TO made the description plan of this review article, and performed manuscript writing and figure charting. SI, KK, and NM made arrangements on the manuscript according their discussions.

### Conflict of interest statement

The authors declare that the research was conducted in the absence of any commercial or financial relationships that could be construed as a potential conflict of interest.
